# Differential Impact of Risk Factors for Women and Men on the Risk of Major Depressive Disorder

**DOI:** 10.1016/j.annepidem.2012.04.011

**Published:** 2012-06

**Authors:** Bauke T. Stegenga, Michael King, Diederick E. Grobbee, Francisco Torres-González, Igor Švab, Heidi-Ingrid Maaroos, Miguel Xavier, Sandra Saldivia, Christian Bottomley, Irwin Nazareth, Mirjam I. Geerlings

**Affiliations:** a Julius Center for Health Sciences and Primary Care, University Medical Center Utrecht, the Netherlands; b Department of Mental Health Sciences, University College London, London, United Kingdom; c Department of Primary Care and Population Health, University College London, London, United Kingdom; d Centro de Investigación Biomedica en Red de Salud Mental (CIBERSAM), Departmental Section of Psychiatry and Psychological Medicine, University of Granada, Granada, Spain; e Department of Family Medicine, University of Ljubljana, Ljubljana, Slovenia; f Faculty of Medicine, University of Tartu, Tartu, Estonia; g Faculdade Ciências Médicas, University of Lisbon, Lisbon, Portugal; h Departamento de Psiquiatra'ıa y Salud Mental, Universidad de Concepción, Concepción, Chile; i Infectious Diseases Epidemiology Unit, London School of Hygiene & Tropical Medicine, London, United Kingdom; j Medical Research Council General Practice Research Framework, London, United Kingdom

**Keywords:** Depression, Sex, Epidemiology, Interaction Contrast, Cohort, CIDI, Composite International Diagnostic Interview, MDD, Major depressive disorder

## Abstract

**Purpose:**

Our aim is to examine which risk factors have a greater impact in women than in men on the risk of major depressive disorder (MDD) and whether factors differ between a possible recurrent MDD and a first onset of MDD.

**Methods:**

Prospective cohort study of general practice attendees in seven countries, who were followed up at 6 and 12 months (predictD). Absolute risk differences (interaction contrast) across sex for onset of DSM-IV MDD after 6 or 12 months of follow-up were estimated for 35 risk factors from 7101 participants without MDD at baseline.

**Results:**

A total of 599 participants (80% female) had an onset of MDD at 6 or 12 months. Most risk factors had a greater impact in women than in men on the risk of MDD and were not restricted to a specific class of risk factors. After we stratified for a history of depressive symptoms, we found that the impact of risk factors across sex was generally stronger on possible recurrent MDD than on a first onset of MDD.

**Conclusions:**

Our findings may partly account for the observed difference in incidence of MDD between men and women.

## Introduction

Major depressive disorder (MDD) is a serious health problem and will be the second leading cause of burden of disease worldwide by 2030 (1). The annual incidence rate of MDD is about 1% to 8%, as shown by population- and primary care–based surveys such as the Stirling County Study, the Lundby Study, the Epidemiologic Catchment Area Study, and the Netherlands Mental Health Survey and Incidence Study (2–9). Epidemiologic research, including ours, has consistently shown that women have greater incidence rates of MDD than men (4, 6, 10, 11). However, the cause of this sex difference remains unclear. Several hypotheses have been put forward (10, 12–14). Some researchers argue that biological factors such as genetic differences may account for the sex difference (10, 15). Others hypothesize that the difference may be ascribed to artifacts involved in the measurement methods used (14). For example, women may report depressive symptoms more often than men, resulting in greater rates of MDD in women (16). Another hypothesis states that psychosocial factors may have a different impact in women than in men (12, 17, 18).

In the light of the latter hypothesis, several psychosocial factors have been studied. Factors like relationship problems, lack of social support, adverse experiences in childhood, and life events may have a greater impact in women than men, thereby increasing their risk for MDD (10, 12, 17). However, most authors who examined risk factors for onset of MDD did not discriminate a first onset of MDD from recurrent MDD (17). In addition, often studies did not have sufficient power to examine the differential impact of risk factors on a first onset of MDD in men and women. Insight into these risk factors may assist physicians and policy makers in determining who is more susceptible to depressive symptoms to prevent the onset of MDD and its associated burden of disease.

Our first aim is to examine which risk factors have a greater impact in women compared with men on the risk of onset of major depressive disorder in a large cohort of primary care attendees. Our second aim is to examine whether these factors are different for those with recurrent MDD compared to those with a first onset of MDD.

## Subjects and Methods

### Study Setting and Design

PredictD is a multicenter prospective cohort study from which a multifactor algorithm was developed to predict risk of onset of major depressive disorder in primary care attendees in six European countries and Chile. This has been described in greater detail elsewhere (7, 11, 19–21). The study was approved by local ethical committees and conducted in seven countries: (1) 25 general practices in the Medical Research Council's General Practice Research Framework, in the United Kingdom; (2) nine large primary care centres in Andalucía, Spain; (3) 74 general practices nationwide in Slovenia; (4) 23 general practices nationwide in Estonia; (5) seven large general practice centres near Utrecht, The Netherlands; (6) two large primary care centres, one in the Lisbon area (urban) and the other in Alentejo (rural), Portugal; and (7) 78 general practices in Concepción and Talcahuano in the Eighth region of Chile.

### Study Participants

Consecutive attendees were recruited (*N* = 10,045) and interviewed between April 2003 and February 2005, and reinterviewed after 6 and 12 months. Exclusion criteria were an inability to understand one of the main languages involved, psychosis, dementia, and incapacitating physical illness. Recruitment differed slightly in each country because of local service preferences. In the United Kingdom and the Netherlands, researchers approached patients waiting for consultations, whereas in the other countries doctors first introduced the study before contact with the research team. All patients gave written informed consent and undertook a research evaluation within two weeks. For the present analyses, we included participants who had no MDD in the 6 months before baseline (*n* = 8517).

### Outcome Measure

A diagnosis of MDD in the preceding 6 months was assessed in all patients at baseline and after 6 and 12 months according to DSM-IV criteria by use of the depression section of the Composite International Diagnostic Interview (CIDI) (22, 23).

### Risk Factors

We selected risk factors for MDD which cover all important areas identified in a systematic review of the literature performed for the predictD study (24). The risk factors, which were also used in our previous work, were assessed at baseline using risk factor questionnaires, unless otherwise stated below (7, 11).

The following risk factors were included in this study:

#### Sociodemographic or Personal

• Age (1),
• Education level (2),
• Marital status (3),
• Employment (4),
• Ethnicity (5),
• Born in the country of residence or abroad (6),
• Religious or spiritual beliefs (7), and
• The presence of long-standing physical illness (8).

#### Psychiatric Comorbidity and Function

• Hazardous alcohol use (9) using the WHO's AUDIT questionnaire (score cut off below 8 or equal and greater);
• Questions on whether the respondent had ever had an alcohol problem (10) or treatment for same;
• Ever used recreational drugs (11), adapted from the relevant sections of the CIDI;
• Anxiety (12) and panic (13) symptoms in the previous 6 months determined by relevant sections of the Patient Health Questionnaire (PHQ); and
• Physical (14) and mental function (15) as assessed by the Short-Form 12.

#### Adverse Experiences in Childhood and Life Events

• Physical and/or emotional abuse (16) and sexual abuse (17) experienced during childhood, and
• Major life events (18) in the preceding 6 months as determined by the List of Threatening Life Experiences Questionnaire.

#### Work, Living, and Environment

• Whether their occupation required specialized knowledge (19).
• Controls, demands, and rewards for paid and unpaid work in the preceding 6 months were estimated by an adapted version of the job content instrument. Participants were categorised as feeling in control in paid (20) or unpaid work (21); as experiencing difficulties without support in paid or unpaid work (22); and experiencing distress without feeling respect for their paid or unpaid work (23).
• Financial strain, which was a single question commonly used in government and other UK social surveys (24).
• Living alone or with others (25).
• Owner–occupier accommodation (26).
• Whether satisfaction with their living conditions was present (27).
• Satisfaction with neighbourhood (28) and perception of safety inside/outside of the home (29) were assessed using questions from the Health Surveys for England.
• Experiences of discrimination (30) on the grounds of sex, age, ethnicity, appearance, disability, or sexual orientation.

#### Family and Friends

• Brief questions on the quality of sexual (31) and emotional (32) relationships with a partner were adapted from a standardized questionnaire;
• The presence of serious physical, psychological or substance misuse problems, or any serious disability in people who were in close relationship to participants (33);
• Difficulties in getting on with people and maintaining close relationships were assessed using questions from a social functioning scale (34);
• Family psychiatric history: serious psychological problems in first-degree family members requiring pharmacologic or psychological treatment in primary or secondary care (35);
• Suicide in first-degree relatives (36); and
• The adequacy of social support from family and friends (37).

Most risk factors were binary; where they were not, they were converted into binary variables as this was needed for the analysis. Two variables (physical and mental function) that were originally continuous were categorized as being below or above the median score. Where a variable had more than two categories, it was recoded so that the category with the greatest prevalence of MDD was compared with the remaining categories combined. Age was analyzed both as a continuous variable and categorized into tertiles, and life events was categorized into 0, 1, and 2 or more events.

### Data Analysis

First, we calculated characteristics for men and women without MDD in the 6 months before baseline. Variables with >20% missing data were dropped from further analysis. Next, for each risk factor we calculated which percentage of men and women had an onset of MDD at 6 or 12 months of follow-up. Onset was defined as a diagnosis of MDD between baseline and 6 months or between 6 and 12 months of follow-up. In women and men, we calculated the absolute risk difference between those with the risk factor compared with those without the risk factor. We calculated absolute risks rather than relative risks as we were interested in the impact of risk factors across sex. To estimate whether the impact of a risk factor was different in women than in men, we calculated the interaction contrast (25) by hand and by using the GLM procedure in PASW version 17.

The interaction contrast is used to compare the risk difference between men and women given the risk factor. Consider the risk factor education. Suppose there are 100 women with lower levels of education and 100 women with higher levels of education. If 10 of the 100 women with lower levels of education become depressed and 5 of the 100 women with higher levels of education become depressed, then the risk difference among women is 10/100 – 5/100 = 5%. Suppose we obtain a risk difference of 3% in men. The difference in risk differences between women and men (i.e., the interaction contrast) is then 5% – 3% = 2%. In this example, the impact of lower levels of education on the risk of becoming depressed is 2% greater for women than for men.

We calculated an accompanying 95% confidence interval for the interaction contrast (26). Note that 1/risk difference = the number needed to harm, i.e., how many patients need to be exposed to a risk factor to cause harm in one patient that would not otherwise have been harmed. In an additional step, age, level of education, number of recent negative life events, and country were added to the models to control for potential confounding. In a subsequent analysis, each risk factor that was significantly different (*p* < .05) in risk for women compared with men was entered in a model that also contained age, sex, level of education, number of recent negative life events, country and all other risk factors with *p* of .05 to .10.

To examine whether the impact of risk factors across sex on possible recurrent MDD was different than on a first onset of MDD at 6 or 12 months of follow-up, we repeated all analyses in strata of a lifetime history of depressive symptoms prior to baseline. A lifetime history of depressive symptoms was ruled out if the two core symptoms of the lifetime CIDI depression section were absent. If one or two of the core symptoms were present, participants were considered to have a lifetime history of depressive symptoms before baseline. All analyses were complete-case analysis because missing data were few. The Hosmer and Lemeshow test showed adequate goodness-of-fit of the models. Analyses were performed using PASW version 17 (IBM SPSS Statistics).

## Results

**Figure 1:**
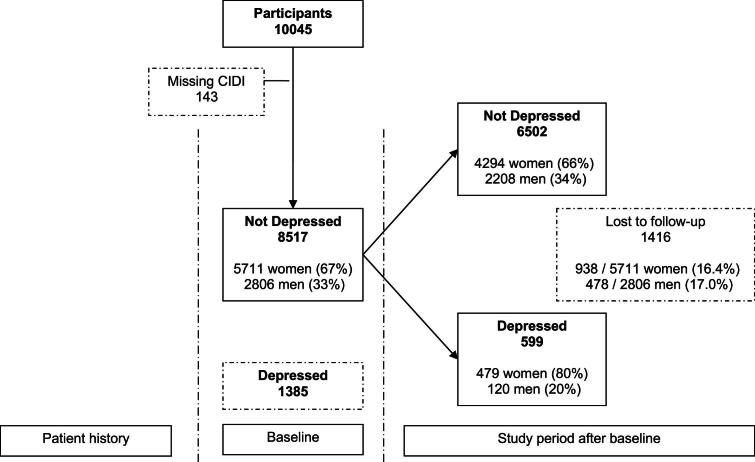
Flowchart of participants without MDD in the 6 months before baseline who have an onset of MDD at 6 or 12 months of follow-up.

The characteristics of the 8517 participants without MDD in the 6 months before baseline (5711 women, mean age 46 years with standard deviation 16 and 2806 men, mean age 51 years with standard deviation 17) are presented in Table 1. Most risk factors were more common among women. Of the 8517 participants without MDD in the 6 months before baseline, 7101 (83.3%) had full data throughout the study (Fig. 1 or Appendix 1). Attrition rates were similar for men (17.0%) and women (16.4%). Eight percent (*N* = 599) had an onset of MDD at 6 or 12 months of follow-up, of whom 479 (80%) were female and 120 male.

Twenty-eight risk factors (80.0%) had a greater impact in women than in men on the risk of onset of MDD at 6 or 12 months of follow-up (Table 2). The risk factors lack of control in paid work and dissatisfied with partner were dropped from further analysis because they had more than 20% missing data. The following risk factors had a significantly greater impact in women: lower levels of education, non-European ethnicity, religious or spiritual, lifetime alcohol problem, anxiety syndrome, two or more recent life events, financial strain, a neighborhood perceived as not being safe, and problems with someone close. In men, the following risk factors had a significantly greater impact on the risk of onset of MDD at 6 or 12 months of follow-up: a nonprofessional occupation and living alone. The results were similar when the models were adjusted for age, level of education, number of recent negative life events, and country (data available on request). Lower levels of education, non-European ethnicity, religious or spiritual, two or more recent life events, financial strain, and a neighborhood perceived as not being safe still had a greater impact in women than in men when all other (borderline) significantly different risk factors were added to the models (data available on request).

**Figure 2:**
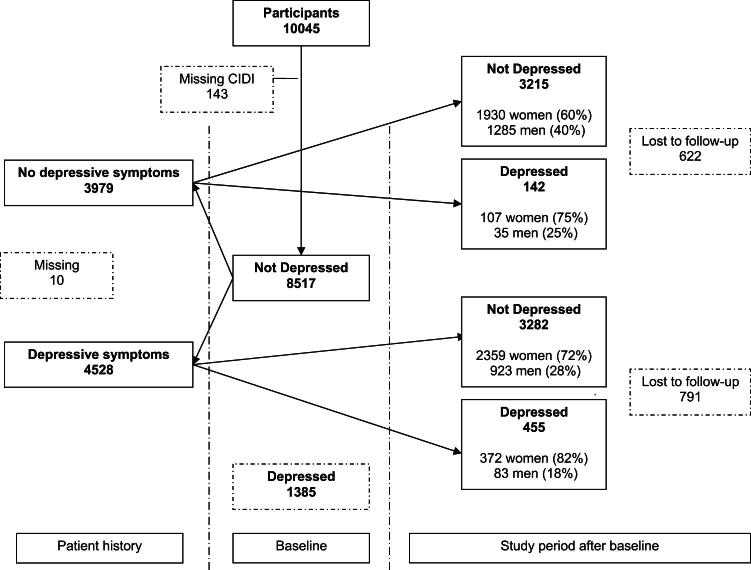
Flowchart of participants without MDD in the 6 months before baseline who have a first onset of MDD or possible recurrent MDD at 6 or 12 months of follow-up.

Of the participants who had no MDD in the 6 months before baseline, 3979 had no lifetime history of depressive symptoms, and 4528 had a lifetime history of depressive symptoms (Fig. 2 or Appendix 2). Of those with no lifetime history of depressive symptoms, 3357 participants had full data throughout the study of whom 142 (4.2%) had a first onset of MDD at 6 or 12 months of follow-up (107 women and 35 men). Of those with a lifetime history of depressive symptoms, 3737 participants had full data throughout the study of whom 455 (12.2%) had a possible recurrent MDD at 6 or 12 months of follow-up (372 women and 83 men). The distribution of women and men with or without a lifetime history of depressive symptoms was fairly similar across all countries (Table 3). The age and sex distribution were similar in those with a recurrent MDD at 6 or 12 months of follow-up and those with a first onset of MDD at 6 or 12 months of follow-up: mean age was 49 years, and more than two-thirds were female. The impact of risk factors on recurrent MDD at 6 or 12 months of follow-up was generally comparable with the impact of risk factors on MDD before stratification for a lifetime history of depressive symptoms before baseline, although the impact of some risk factors became greater than in the whole population (e.g., lower levels of education) and some risk factors lost statistical significance (e.g., a lifetime alcohol problem; see also Table 2).

When we considered those with a possible first onset of MDD at 6 or 12 months of follow-up, most risk factors had a greater impact in women than in men, although the impact of risk factors was generally weaker than on recurrent MDD at 6 or 12 months of follow-up. For example, lower levels of education had a greater impact in women than in men on recurrent MDD but not on a first onset of MDD. A neighborhood that was perceived as not being safe had a greater impact in women than in men on both recurrent MDD and on a first onset of MDD. In contrast, living alone had a greater impact in men than in women on recurrent MDD as well as on a first onset of MDD. Dissatisfied with living condition had a greater impact on recurrent MDD in women but a greater impact on a first onset of MDD in men. The results were similar when the models were adjusted for age, level of education, number of recent negative life events and country (data available on request).

## Discussion

In this large-scale, cross-national study three main observations emerged: (1) most risk factors studied had a greater impact in women than in men on the risk of onset of MDD at 6 or 12 months of follow-up, independent of confounding factors; (2) risk factors that had greater impact in women were not restricted to a specific class of risk factors but varied across different groups of risk factors; and (3) the impact of risk factors across sex was generally stronger on recurrent MDD at 6 or 12 months of follow-up than on a first onset of MDD at 6 or 12 months of follow-up.

A body of research has examined sex differences in risk factors for onset of MDD (10, 12, 17). However, most studies did not discriminate a first onset of MDD from recurrent MDD, which makes it difficult to make direct comparisons (17). The finding that most risk factors studied had a greater impact in women than in men suggests that women are at greater risk of becoming depressed when a risk factor is present. It has been suggested that women may have greater biologic vulnerability to onset of MDD (10). Although women were generally more likely to be exposed to risk factors than men, as most risk factors were more common among women, our findings suggest that women may also be more likely to get affected by presence of risk factors than men.

The risk factors that had greater impact in women than in men were not restricted to a specific class of risk factors, such as sociodemographic or personal factors. However, two risk factors that had the strongest impact had a greater impact on recurrent MDD at 6 or 12 months of follow-up as well as on a first onset of MDD at 6 or 12 months of follow-up: a neighborhood that was perceived as not being safe in women and living alone in men. In addition, being dissatisfied with living condition was the third factor that had the strongest impact on recurrent as well as on a first onset of MDD, although it had a greater impact in women on recurrent MDD and in men on a first onset of MDD. The relationship between poor neighbourhood conditions and depressive symptoms has been well established (27). For example, poverty status may be associated with first onset of MDD in a 1-year period (28). Our study is the first to show that a neighborhood that was perceived as not being safe had a stronger impact in women than in men to become depressed, irrespective of whether a lifetime history of MDD before baseline was considered. Living alone had a significantly greater impact in men than in women on recurrent MDD as well as on a first onset of MDD. Studies among aged populations found that living alone is associated with MDD and an increased risk of suicide in men (29). The few adult population based studies reported that living alone may have a stronger impact on mental health in men than in women (30, 31). It could be that men who are living alone become more easily isolated than women as the latter may maintain more active social ties to family and friends (32). Isolation may in turn lead to onset of depressive symptoms. Men with lower status jobs who are isolated, form a recognizable group for social or health interventions.

The observation that being dissatisfied with living condition had a greater impact in women on recurrent MDD but in men on a first onset of MDD may suggest that in men it could be a real risk factor for a first onset of MDD, whereas in women dissatisfaction with living condition may have been caused by their lifetime history of depressive symptoms prior to baseline. It could be that previous depressive symptoms have sensitized women for perceiving and reporting risk factors (33). It could also be that women living in unsafe areas perceive greater risk of harm than men, even though they may not be at greater risk. Social support such as women's groups, neighborhood watch groups or community policing aimed at supporting women might be helpful to reduce these perceptions of risk.

We observed that the impact of risk factors across sex was generally stronger on recurrent MDD at 6 or 12 months of follow-up than on a first onset of MDD at 6 or 12 months of follow-up. This finding suggests that the strength of impact of risk factors in men and women may be different on recurrent MDD than on a first onset of MDD, which may be in accordance with the kindling hypothesis (33). This hypothesis suggests that susceptibility to a subsequent MDD may alter after onset of MDD as occurrence of a first onset of MDD may largely depend on the level of stress, whereas recurrent MDD may occur independent of stress. Risk factors that had a greater impact across sex on recurrent MDD were comparable with those before stratification for a lifetime history of MDD before baseline. Studies in which the authors examined onset of MDD did not always take a lifetime history of MDD into account. It could be that these studies examined recurrent MDD rather than a first onset of MDD. Our findings suggest that it is important to take a lifetime history of MDD into account when examining risk factors for onset of MDD, and to note the difference between recurrent MDD (i.e., new episode of depressive illness following recovery in those without MDD at baseline and with a lifetime history of MDD prior to baseline) and recurrence of MDD (i.e., new episode of depressive illness following recovery in those with MDD at baseline) (34).

Strengths of our study are that our cohort was large and included participants from six European countries and Chile. We diagnosed MDD by using the *Diagnostic and Statistical Manual of Mental Disorders*, Fourth Edition criteria, and response to follow-up was high in all countries. Because we included lifetime history of MDD data, not only were we able to examine recurrent MDD but also a first onset of MDD from a lifetime perspective. We assessed a wide range of risk factors for MDD which reflect the current state of knowledge (11). Our study was limited by the lower response to recruitment in the United Kingdom and the Netherlands, which possibly occurred because the study was not so obviously endorsed by family doctors compared with the other countries in the study (7).Yet, attrition was low and was not related to sex.

Another limitation is that biologic factors and data on smoking were not available, which could have distorted the impact of risk factors on MDD across sex. For example, increased sensitivity to hormonal changes during the menopausal transition may increase vulnerability to onset of MDD for women and not for men (35). Another potential limitation is that presence of a lifetime history of MDD before baseline was determined by an affirmative answer to either of the two core questions of the CIDI rather than assessment of a full CIDI depression interview. Although we excluded those who had dementia, we cannot rule out the possibility that cognitive impairment may have influenced the recalling of previous episodes of MDD in those who were older. Also, it is possible that general practice effects were present, however we were unable to analyse this as a result of the sample size.

Clinicians need to be aware of the difference in susceptibility and hence the enhanced risk of women with known risk factors for depression being at greater risk of developing depression. Most risk factors studied had a greater impact in women than in men on the risk of onset of MDD at 6 or 12 months of follow-up, independent of confounding factors. In particular, they need to be extra vigilant in women with lower levels of education, non-European ethnicity, with religious or spiritual beliefs, lifetime alcohol problems, anxiety syndrome, two or more recent life events, financial strain, a neighborhood perceived as not being safe, and problems with someone close. On the other hand, for men greater vigilance is required for those in nonprofessional occupations and living alone. Risk factors that had greater impact in women were not restricted to a specific class of risk factors but varied across different groups of risk factors. Finally, in people with a known history of depression, the presence of known risk factors for depression confers greater vulnerability, and it may be reasonable to consider offering relevant interventions before the onset of their illness that have worked in the past for the management of their depression to perhaps prevent a new onset of the illnesses. This will, however, require research evaluation.

In conclusion, most risk factors studied had a greater impact in women than in men on the risk of onset of MDD and were not restricted to a specific class of risk factors. These findings may partly account for the observed difference in incidence between men and women and may assist in the prevention of depressive symptoms and the burden of MDD. Future studies should discriminate a first onset of MDD from recurrent MDD.

## Figures and Tables

**Table 1:** Baseline characteristics for 8517 persons with no major depressive disorder in the 6 months before baseline

	Women (*n* = 5711)	Men (*n* = 2806)
Sociodemographic or personal		
Age in years, mean (SD)∗	47 (16)	51 (17)
Age in years, tertiles∗		
w18–43	2360 (42)	887 (33)
44–58	1680 (30)	800 (30)
59–75	1545 (28)	1021 (38)
Lower education∗	2371 (42)	1232 (44)
Not married/living with partner∗	2046 (36)	718 (26)
Unemployed∗	3090 (54)	1278 (46)
Ethnicity: non-European∗,†	1717 (30)	730 (26)
Immigrant‡	275 (6)	152 (6)
Religious or spiritual∗,†	4385 (78)	1892 (69)
Longstanding physical illness∗	2433 (43)	1311 (47)
Psychiatric comorbidity and function		
Hazardous alcohol use∗	166 (3)	397 (14)
Lifetime alcohol problem∗	180 (3)	358 (13)
Ever used recreational drugs	384 (7)	210 (8)
Other anxiety syndrome∗	287 (5)	64 (2)
Panic syndrome∗	316 (6)	85 (3)
SF-12 Physical function below median∗	2803 (49)	1252 (45)
SF-12 Mental function below median∗	2696 (47)	1005 (36)
Adverse experiences and life events		
Physical or emotional abused∗	1135 (20)	443 (16)
Sexual child abused∗	325 (6)	44 (2)
Recent negative life events		
No	1693 (30)	852 (31)
One	1718 (30)	855 (31)
Two or more	2286 (40)	1091 (39)
Work, living and environment		
Occupation: nonprofessional∗,§	630 (13)	450 (18)
Lack of control in paid work¶	1135 (42)	660 (41)
Lack of control in unpaid work†	1144 (20)	554 (20)
Difficulties at work without support	641 (11)	283 (10)
Distress at work without respect∗,†	654 (12)	205 (8)
Financial strain∗	1840 (32)	767 (28)
Living alone∗	582 (10)	225 (8)
Accommodation: not owned∗	1387 (24)	615 (22)
Dissatisfied with living condition∗,‖	801 (15)	350 (13)
Dissatisfied with neighborhood∗	979 (17)	425 (15)
Neighbourhood perceived not safe∗	430 (8)	150 (5)
Discrimination∗	546 (10)	228 (8)
Family and friends		
Dissatisfied with overall sex life†	778 (14)	411 (15)
Dissatisfied with partner∗,#	518 (12)	229 (10)
Problems with someone close∗	2178 (38)	865 (31)
Difficulties in getting along with people†	378 (7)	155 (6)
Family history of psychiatric disorder∗	1803 (32)	728 (26)
Suicide in first-degree relatives	159 (3)	74 (3)
Social support below median∗	2391 (42)	1337 (48)

SF-12 = Short Form-12. All variables are presented as N (%), unless otherwise stated above.

∗ *p* < .05. All variables have ≤1% missing data, except:

† Missing data = 2%–3%.

‡ Missing data = 11%.

§ Missing data = 12%.

‖ Missing data = 7%.

¶ Missing data = 49%.

# Missing data = 23%.

**Table 2:** Differential impact of risk factors among women compared with men on the risk of onset of major depressive disorder at 6 or 12 months of follow-up

	*N*	Risk for MDD in women *with* the risk factor %	Risk difference in women *with* and *without* RF %	Risk for MDD in men *with* the risk factor %	Risk difference in men *with* and *without* RF %	Onset of MDD at follow-up IC_unadjusted_		Possible recurrent MDD at follow-up, *n* = 3737 IC_unadjusted_		Possible first onset of MDD at follow-up *n* = 3357 IC_unadjusted_	
						%	95% CI	%	95% CI	%	95% CI
Sociodemographic or personal											
Age in years, tertiles∗	6910										
18–43		9.4	0.9	3.8	−1.4	2.2	−1.2 to 5.6	0.6	−5.2 to 6.4	4.1†	0.7 to 7.5
44–58		12.5	4.0	7.1	1.9	2.0	−1.5 to 5.5	0.6	−5.3 to 6.5	2.7	−0.9 to 6.3
Lower education	7052	14.1	6.8	5.9	1.5	5.3†	2.5 to 8.1	7.2∗	2.4 to 12.0	2.5	−0.3 to 5.4
Not married/living with partner	7079	10.9	1.3	6.8	2.2	−0.8	−3.9 to 2.3	−2.2	−7.5 to 3.1	−0.4	−3.6 to 2.7
Unemployed	7063	11.7	3.5	6.3	2.1	1.5	−1.3 to 4.2	1.7	−3.0 to 6.5	1.0	−1.9 to 3.7
Ethnicity: non-European	7039	13.5	4.8	5.4	0.3	4.5†	1.4 to 7.6	10.3†	4.6 to 16.0	−0.4	−3.3 to 2.7
Immigrant	6239	11.2	0.7	7.1	2.1	−1.4	−7.7 to 4.9	−6.2	−16.6 to 4.3	4.9	−1.8 to 11.5
Religious or spiritual	6981	11.1	4.8	5.1	0.2	4.7†	1.6 to 7.8	7.2†	1.8 to 12.5	2.1	−1.1 to 5.1
Longstanding physical illness	7066	12.5	4.2	7.5	4.4	−0.2	−3.0 to 2.5	−2.1	−6.9 to 2.6	−0.1	−3.0 to 2.7
Psychiatric comorbidity/function											
Hazardous alcohol use	7064	11.5	1.5	4.0	−1.3	2.8	−3.1 to 8.7	4.3	−4.8 to 13.4	−0.6	−7.4 to 6.3
Lifetime alcohol problem	7075	20.8	11.1	9.6	5.2	6.0†	0.4 to 11.6	5.4	−2.8 to 13.7	2.7	−4.7 to 9.9
Ever used recreational drugs	7030	13.5	3.7	6.2	1.1	2.5	−3.1 to 8.2	2.5	−6.3 to 11.2	2.8	−3.6 to 9.1
Other anxiety syndrome	7022	33.9	25.0	21.2	16.5	8.6†	0.2 to 16.9	8.1	−3.3 to 19.6	9.0	−3.5 to 21.6
Panic syndrome	7069	28.0	19.0	21.1	16.5	2.5	−4.8 to 9.9	−0.5	−10.5 to 9.5	9.0	−2.2 to 20.3
SF-12 Physical function below median	7101	12.9	5.6	7.8	4.7	1.0	−1.7 to 3.8	0.0	−4.7 to 4.7	0.8	−2.1 to 3.6
SF-12 Mental function below median	7101	15.0	9.3	9.5	6.7	2.6	−0.2 to 5.4	3.0	−1.7 to 7.7	0.0	−3.1 to 3.0
Adverse experiences/life events											
Physical or emotional abused	7073	16.6	8.3	10.2	6.0	2.2	−1.4 to 5.9	5.0	−0.7 to 10.7	−2.1	−6.2 to 0.2
Sexual child abused	7060	19.3	9.9	17.9	13.0	−3.2	−12.5 to 6.2	0.2	−14.4 to 14.7	−5.8	−16.8 to 5.0
Recent negative life events‡	7082										
One		10.0	3.2	4,9	1.0	2.2	−1.1 to 5.4	2.9	−2.8 to 8.6	−0.1	−3.3 to 3.2
Two or more		14.8	8.0	7.3	3.4	4.5†	1.2 to 7.9	5.6∗	0.0 to 11.3	0.7	−2.7 to 4.2
Work, living and environment											
Occupation: nonprofessional	6191	6.8	−4.4	5.9	0.8	−5.2†	−9.3 to −1.2	−5.8	−12.7 to 1.0	−3.9	−8.0 to 0.3
Lack of control in unpaid work	6962	11.6	1.9	6.4	1.6	0.2	−3.2 to 3.7	−2.2	−7.9 to 3.5	2.4	−1.2 to 6.1
Difficulties at work without support	7026	17.8	8.6	10.0	5.4	3.2	−1.3 to 7.8	3.4	−3.9 to 10.6	1.2	−3.9 to 6.3
Distress at work without respect	6989	18.3	9.3	11.9	7.3	2.0	−3.0 to 7.0	2.9	−4.8 to 10.6	−1.0	−6.9 to 4.8
Financial strain	7082	15.9	8.5	7.8	3.5	5.0†	2.0 to 8.1	6.8†	1.7 to 11.9	0.8	−2.4 to 4.0
Living alone	7101	8.6	−1.6	11.8	7.2	−8.8†	−13.7 to −3.8	−11.0†	−18.9 to −3.0	−7.7†	−13.1 to −2.3
Accommodation: not owned	7062	11.8	2.3	6.3	1.4	0.9	−2.4 to 4.3	1.1	−4.6 to 6.8	0.4	−3.0 to 3.8
Dissatisfied with living condition	6648	13.5	4.9	6.5	1.9	3.0	−1.1 to 7.0	7.3†	0.6 to 14.1	−5.0∗	−9.3 to −0.8
Dissatisfied with neighborhood	7095	14.0	4.8	7.8	3.1	1.7	−2.1 to 5.5	5.5	−0.7 to 11.7	−3.2	−7.3 to 0.9
Neighborhood perceived not safe	7095	20.2	11.0	5.3	0.1	10.8†	4.8 to 16.8	11.0†	1.4 to 20.7	10.3†	3.8 to 16.8
Discrimination	7073	18.7	9.6	9.9	5.4	4.1	−0.7 to 9.0	3.9	−3.6 to 11.3	2.6	−3.0 to 8.2
Family and friends											
Dissatisfied with overall sex life	6890	12.7	3.0	8.3	3.7	−0.7	−4.6 to 3.2	−3.4	−9.6 to 2.8	2.2	−2.2 to 6.6
Problems with someone close	7066	13.5	5.6	6.4	2.0	3.6†	0.7 to 6.5	4.1	−0.8 to 9.1	2.5	−0.6 to 5.5
Difficulties in getting along with people	6976	14.8	5.0	8.7	3.7	1.3	−4.6 to 7.2	−2.2	−11.1 to 6.6	4.1	−2.9 to 11.1
Family history of psychiatric disorder	7027	13.4	4.9	8.2	4.1	0.7	−2.4 to 3.8	0.1	−5.0 to 5.1	1.1	−2.2 to 4.5
Suicide in first-degree relatives	7036	12.5	2.5	5.6	0.5	2.1	−6.8 to 11.0	−1.8	−16.0 to 12.5	5.0	−5.0 to 14.9
Social support below median	7035	11.1	1.8	5.5	0.7	1.2	−1.6 to 3.9	1.1	−3.7 to 5.9	0.8	−2.0 to 3.6

CI = confidence interval; IC_unadjusted_ = risk difference of the interaction contrast between women and men (unadjusted); MDD = major depressive disorder; RF = risk factor; Risk difference = risk difference between those with the risk factor compared with those without the risk factor; SF–12 = Short Form 12. Note that a positive IC indicates a greater impact in women, whereas a negative IC indicates a greater impact in men

∗ Reference category is persons ages 59–75 years.

† Significant interaction on an additive scale.

‡ Reference category is no recent life events.

**Table 3:** The distribution of women and men with or without a lifetime history of depressive symptoms for all countries, in those with no major depressive disorder in the 6 months before baseline

	*n*	Women without a history of depressive symptoms %	Women with a history of depressive symptoms %	Men without a history of depressive symptoms %	Men with a history of depressive symptoms %	*p*-value
United Kingdom	1131	25	41	14	20	.20
Spain	1011	20	49	14	17	<.001
Slovenia	1050	29	34	22	16	<.001
Estonia	923	29	43	16	12	<.001
Netherlands	1077	30	33	23	15	<.001
Portugal	1008	26	39	19	16	<.001
Chile	2317	34	37	19	11	<.001
Total	8517	28	39	18	15	

Percentages may not add up to 100% because of rounding.
